# Bioinformatics analysis to screen key prognostic genes in the breast cancer tumor microenvironment

**DOI:** 10.1080/21655979.2020.1840731

**Published:** 2020-11-08

**Authors:** Qian Ye, Xiaowen Han, Zhengsheng Wu

**Affiliations:** Department of Pathology, School of Basic Medicine, Anhui Medical University, Hefei, China

**Keywords:** Breast cancer, tumor microenvironment, prognosis, bioinformatics analysis

## Abstract

Increasing evidence has shown that the tumor microenvironment (TME) plays an important role in tumor occurrence and development and can also affect patient prognosis. In this study, we screened key prognostic genes in the breast cancer (BC) TME by analyzing the immune and stromal scores of tumor samples to detect differentially expressed genes (DEGs) and also constructed a TME-related prognostic model. First, we obtained mRNA-Seq and related clinical information for patients with BC from The Cancer Genome Atlas (TCGA) and calculated the stromal and immune scores of tumor tissues using the ESTIMATE algorithm. Next, we performed functional enrichment analysis and generated protein–protein interaction networks from the DEGs that were highly related to the TME. Finally, Cox proportional hazards regression analysis was performed on BC datasets from TCGA, and analyses were conducted on infiltrating immune cells and the human protein atlas. Together, these analyses indicated that the *KLRB1* and *SIT1* genes could be used as independent prognostic factors for BC, while risk score, age, and clinical stage could be used as prognostic factors. In summary, we found that the prognosis of BC is closely related to immune regulation in the TME.

## Graphical abstract

**Figure:**
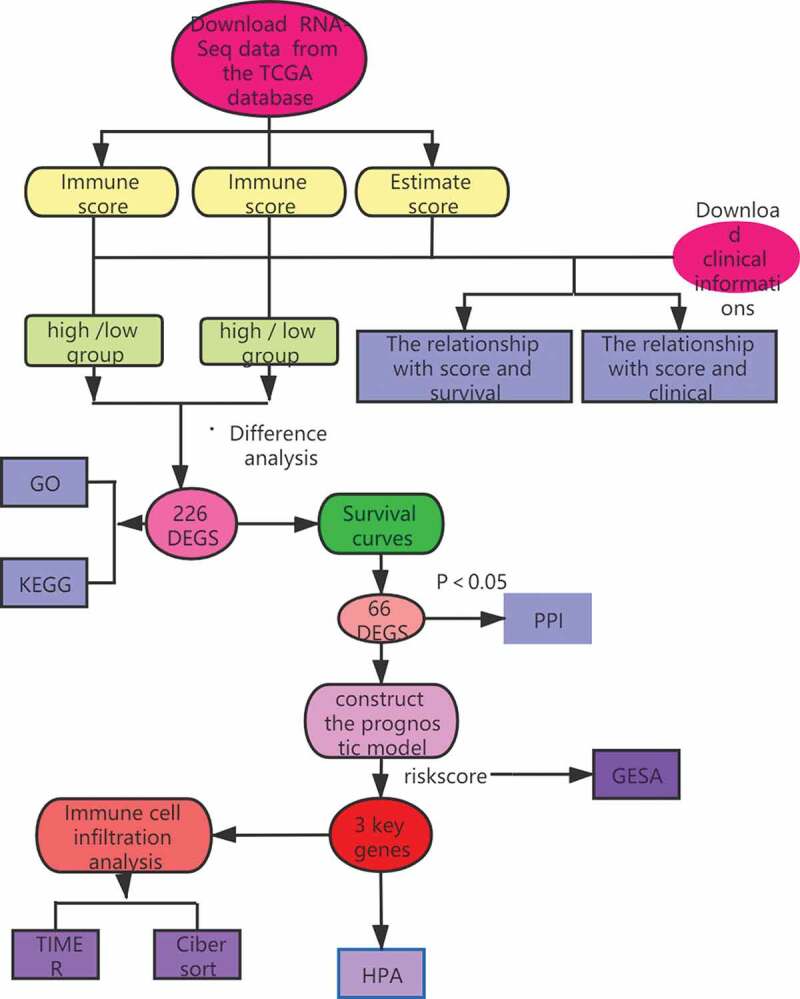

## 1. Introduction

Breast cancer (BC) is the second most common cancer after lung cancer and is a leading cause of cancer-related death among women [1]. BC is a complex and heterogeneous disease with considerable variation in clinical manifestation, morphological and molecular properties, and treatment response [2].

The internal environment of a tumor is known as the tumor microenvironment (TME) and includes both cellular and non-cellular components that have a significant effect on patient prognosis [3]. The cellular components of the TME are considered to be markers of cancer regulation and exert important effects on tumor proliferation, angiogenesis, invasion and metastasis, and chemotherapy resistance [4]. These include stromal cells, immune cells, blood vessels, lymphatic vessels, extracellular matrix (ECM), secreted proteins, RNA, and small organelles [5]. Two-way communication between cells and their microenvironment is crucial for normal tissue homeostasis and tumor growth [6]; however, studies have shown that the TME affects multiple stages of disease development, particularly local drug resistance, immune escape, and distant metastasis [7].

Tumorigenesis and cancer progression are complex and dynamic processes, and considerable evidence has shown that the TME participates in the progression and metastasis of various types of cancer, including BC [8]. Unlike tumor cells, stromal cells in the TME are genetically stable and have therefore become an attractive therapeutic target for reducing drug resistance and the risk of tumor recurrence [9]. A variety of nonmalignant cell types in the TME can also affect the occurrence, development, metastasis, and treatment response of BC; however, the majority of the genetic mechanisms that contribute toward patient outcomes remain unclear [10].Previous studies have demonstrated that the proliferation and metastasis of tumor cells are affected by immune cells and their mechanisms of action [11], including tumor-associated macrophages (TAMs), myeloid-derived suppressor cells (MDSCs), CD4 + Th1 cells, CD8 + T cells, regulatory T cells (Tregs), and TH17 cells [12]. Therefore, the TME and its infiltrating immune cells, cytokines, and growth factors play key roles in regulating BC [13]and provide potential avenues for the development of first-line clinical interventions.

In this study, we screened key prognostic genes in the TME of BC by analyzing the immune and stromal scores of tumor samples to detect differentially expressed genes (DEGs) and constructed a TME-related prognostic model.

## 2. Materials and methods

### 2.1. Data acquisition and processing

We obtained the RNA-Seq data (HTSeq-FPKM) for patients with BC (lobular and ductal tumors) from TCGA database (http://portal.gdc.cancer.gov/), including (gdc_download_20200915_123045.956595, gdc_manifest_20200915_123011, metadata.cart.2020–09-15). Data were organized using perl software (http://www.perl.org/).

### 2.2. ESTIMATE

The immune and stromal scores for each BC sample were acquired using the ‘ESTIMATE’ and ‘limma’ packages in R (version 4.0.2). The estimation algorithm was used to compute the matrix and immune scores of BC tissues and to divide the samples into high (> median) and low (< median) scoring groups.

### 2.3. Identification of DEGs

The ‘limma Bioconductor’ software package in R (version 4.0.2) was used to identify DEGs and divide samples into high/low immune and stromal score groups based on the following cutoff conditions: |log2 fold change (log2FC)| > 1.0, false discovery rate (FDR) < 0.05. A Venn diagram was used to compare the up- and down-regulated intersecting genes related to immune/stromal score [14]. The ‘pheatmap’ software package in R (version 4.0.2) was used to generate heatmaps [15].

### 2.4. Functional enrichment analysis

The ‘clusterProfiler’, ‘enrichplot’, and ‘ggplot2’ software packages in R (version 4.0.2) were used to perform Gene Ontology (GO) and Kyoto Encyclopedia of Genes and Genomes (KEGG) enrichment analyses. GO has three independent branches: molecular function (MF), biological process (BP), and cellular component (CC). The KEGG database facilitates the systematic analysis of the intracellular metabolic pathways and functions of gene products. An FDR of <0.05 was defined as statistically significant [16].

### 2.5. Survival curves

Kaplan-Meier (K-M) survival curves were plotted using the ‘survival’ package in R (version 4.0.2) to analyze the relationship between DEG expression and the overall survival of patients with BC. *P* values of <0.05 were considered statistically significant.

### 2.6. Protein–protein interaction (PPI) network analysis

STRING (https://string-db.org/) is a database that provides a function for predicted protein interactions, in which each PPI has one or more ‘scores’ that indicate the confidence in the interaction based on the available evidence. This score ranges from 0 to 1, with 1 being the highest possible confidence. The interaction relationships between the intersecting genes were acquired by PPI network analysis in STRING. Core genes were identified using the CytoHubba Cytoscape plug-in with the highest confidence (0.40) as a threshold.

### 2.7. Prognostic model construction

To simultaneously analyze the effect of many factors on overall survival (OS) we performed Cox regression analysis on BC patient samples in the TCGA database after excluding patients with no available (NA) survival time to obtain DEGs that affected patient prognosis. *P* values of <0.05 were considered statistically significant. A risk scoring formula was established based on the analysis results.

### 2.8. Gene set enrichment analysis (GSEA)

The selected KEGG gene set was downloaded from the MSigDB database and GSEA (version 4.0.3) was performed to explore the potential molecular mechanisms in the high- and low-risk groups and to acquire pathways for up- and down-regulation. An FDR of <0.05 was considered statistically significant.

### 2.9. Immune cell infiltration analysis

Using the TIMER database (https://cistrome.shinyapps.io/timer/), we retrieved correlations between DEG expression and immune infiltration levels. To estimate differences in the infiltration of 22 immune cell types between the low- and high-risk groups, CIBERSORT (https://cibersort.stanford.edu/about.php) was used to accurately estimate the immune cell components in tumor tissues.

### 2.10 Human protein atlas (HPA)

We obtained an immunohistochemistry expression graph of related genes from the HPA database (https://www.proteinatlas.org/). Multiple genes are differentially expressed in cancer, and many of these have an effect on the OS of patients.

## 3. Results

### 3.1. Effect of immune and stromal scores in patients with BC

The RNA-Seq data and clinical information for 1,049 patients with BC were downloaded from TCGA and tumor samples were evaluated using the ESTIMATE algorithm. Stromal scores ranged from −2070.44 to 2099.46, immune scores ranged from −1188.52 to 3661.56, and the ESTIMATE score ranged from −1188.52 to 3661.56. According to the median scores, all 1049 samples were divided into high- and low-score groups, and the relationships between each score and clinical characteristics were analyzed. The OS of patients with BC was significantly and positively correlated with a higher immune score (*P* = 0.015; Figure 1a); however, there were no significant differences between stromal scores and ESTIMATE scores (Figure 1b,c). Patients in the low-age group were found to always show higher scores (Figure 1d-f), while clinical stage displayed a significant negative association with immune score (*P* = 0.022; Figure 1g) but the stromal score was not statistically significant (*P* = 0.23; Figure 1h). No other clinical factors displayed clear statistically significant effects (supplementary figure 1). These results indicating that stromal and immune scores of BRCA were positive factors in patient prognosis.

**Figure 1.**
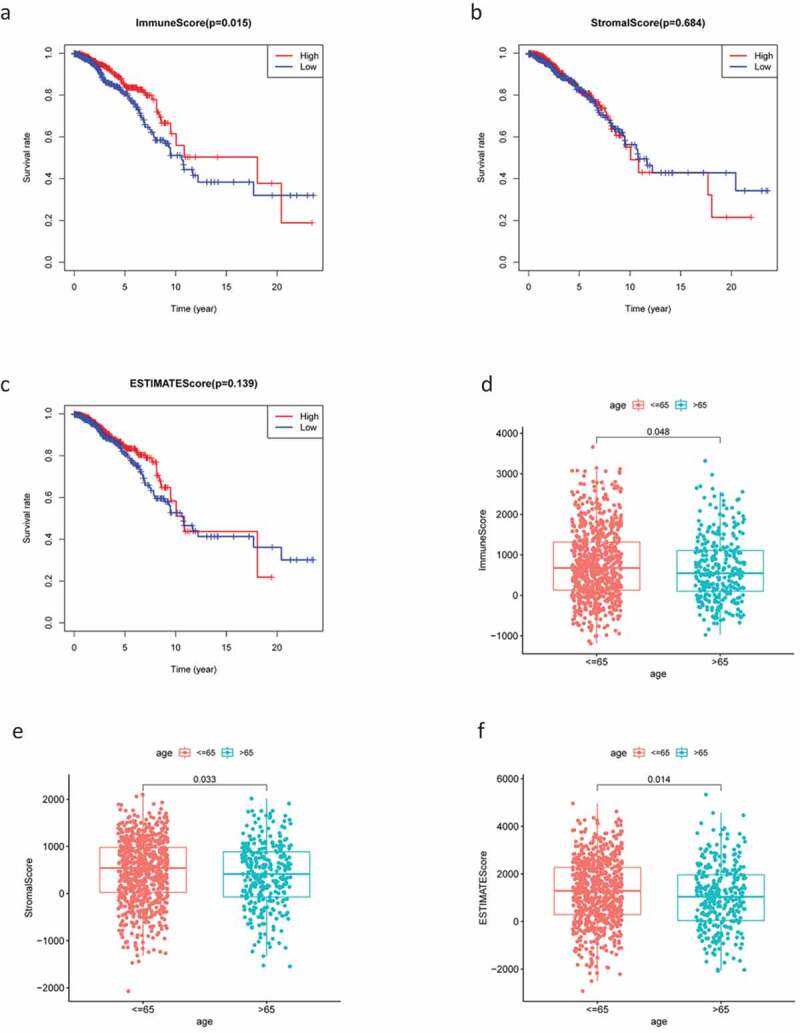
Analysis of correlations between clinical information and stromal/immune scores. Distribution of immune scores according to clinical OS (a), age (d) and stage (g). Distribution of stromal scores according to OS (b), age (e) and stage (h). Distribution of ESTIMATE scores according to OS (c), age (f) and stage (i). OS: overall survival

**Figure 1.**
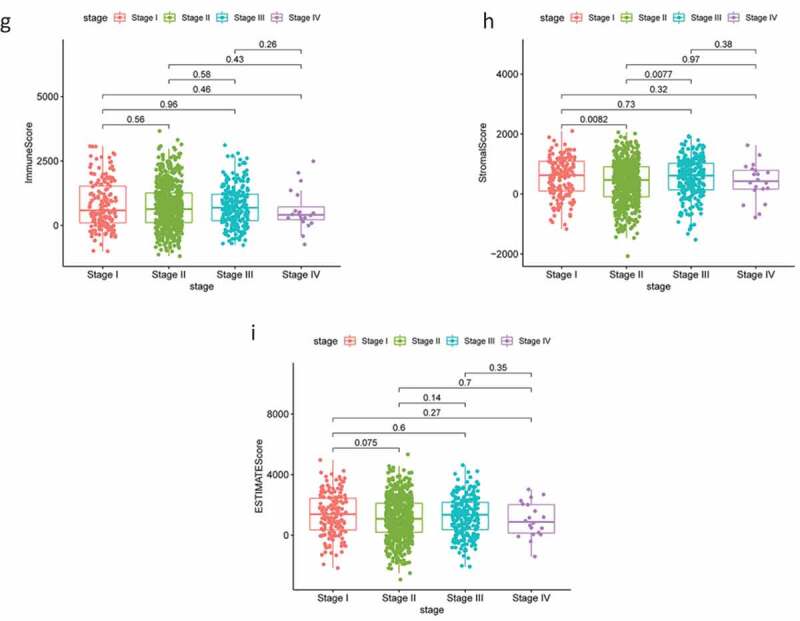
(Continued)

### 3.2. Identification of DEGs

By comparing the gene expression levels of the high- and low-scoring groups, we screened DEGs with |log2FC| > 1.0 and FDR < 0.05, and displayed them as a heat map (Figure 2a,b). Statistical analysis identified 535 significantly upregulated and 77 significantly downregulated DEGs in the immune score group, as well as 452 significantly up-regulated and 108 significantly down-regulated genes in the stromal score group. These genes related to immune and stromal scores were visualized using Venn plots (Figure 2c,d).

**Figure 2.**
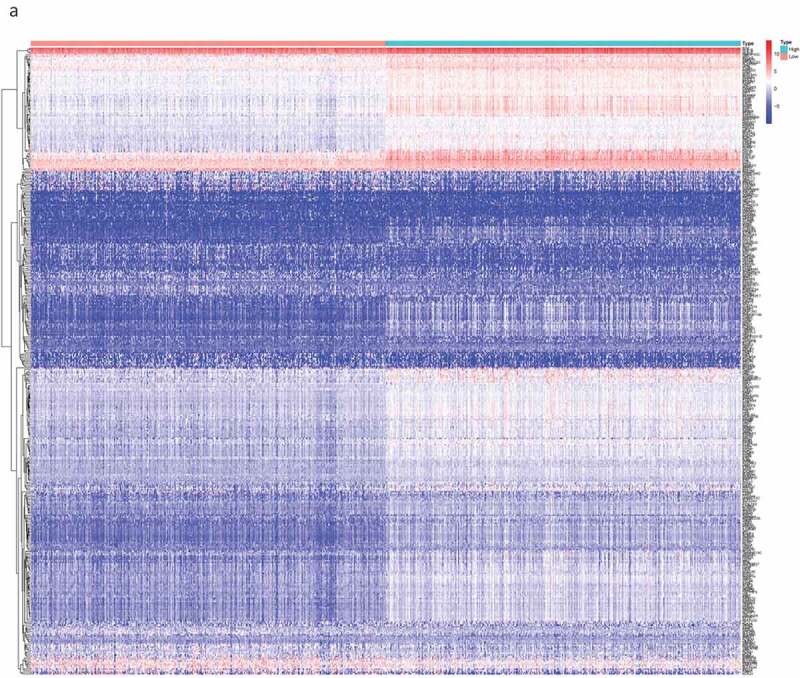
The DEGs between high and low stromal/immune score groups and the overlapping genes related to both stromal and immune score. The heatmap shows the DEGs between the high immune score group and the low immune score group (a). The DEGs between the high stromal score group and the low stromal score group (b). The color indicates the fold change of gene expression; the greater the change is, the darker the color (red is up, blue is down). The Venn diagram shows the upregulated genes related to both immune score and stromal score (c). Downregulated genes related to both immune score and stromal score (d). DEGs: differentially expressed genes

**Figure 2.**
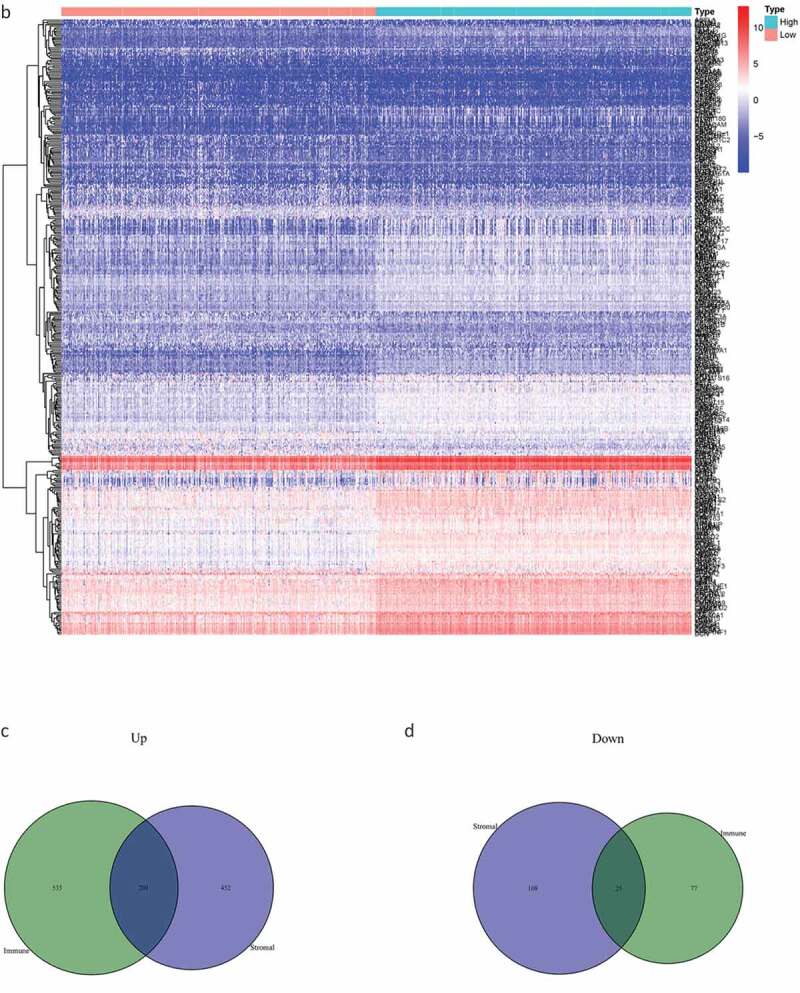
(Continued)

### 3.3. Functional enrichment analysis

Next, we conducted a functional enrichment analysis on 226 significant DEGs. GO analysis (Figure 3a) revealed that the top five enriched BP terms were ‘alpha−beta T cell activation’, ‘mononuclear cell proliferation’, ‘lymphocyte proliferation’, ‘adaptive immune response based on somatic recombination’, and ‘lymphocyte-mediated immunity’. The top five enriched CC terms were ‘external side of plasma membrane’, ‘secretory granule membrane’, ‘tertiary granule’, ‘specific granule’, and ‘ficolin−1−rich granule membrane’. The top five enriched MF terms were ‘immune receptor activity’, ‘cytokine receptor activity’, ‘chemokine binding’, ‘C−C chemokine binding’, and ‘C − C chemokine receptor activity’. The Circos plots (Figure 3b) showed that the GO terms were mainly related to α-β T cell activation, lymphocyte differentiation, the regulation of T cell activation, T cell activation, and T cell differentiation.

**Figure 3.**
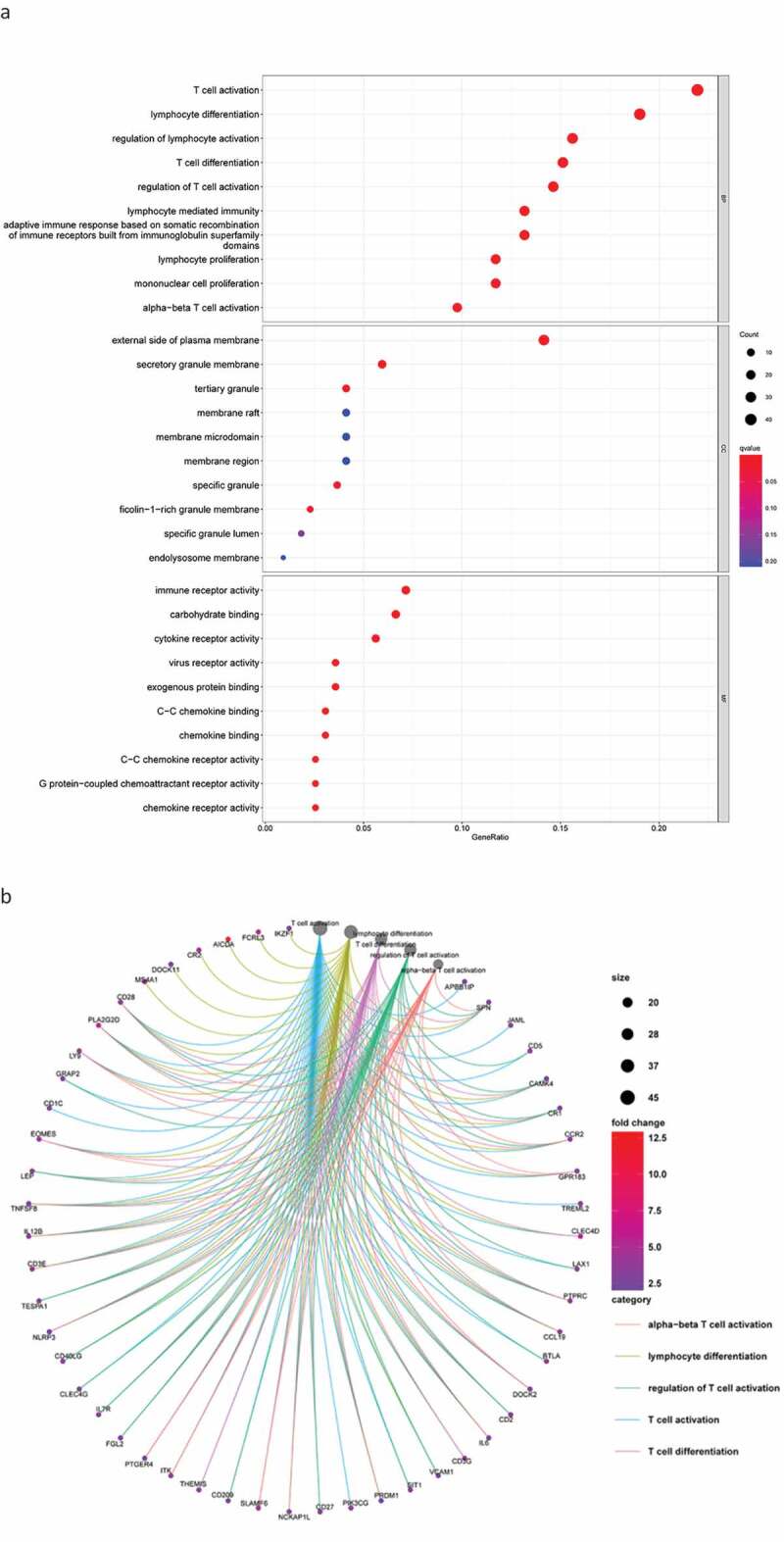
DEG functional enrichment analysis results. (a) GO enrichment results show the top 10 BP terms, CC terms and MF terms. (b) The circos plots show the enrichment relationship between genes and the main enriched terms in GO. (c) The KEGG enrichment results show the 13 paths. (B) The circos plots show the enrichment relationship between genes and the main enriched terms in KEGG. DEG: differentially expressed gene. GO: gene ontology. MF: molecular function, BP: biological process, CC: cellular component. KEGG: Kyoto City Encyclopedia of Genes and Genomes

**Figure 3.**
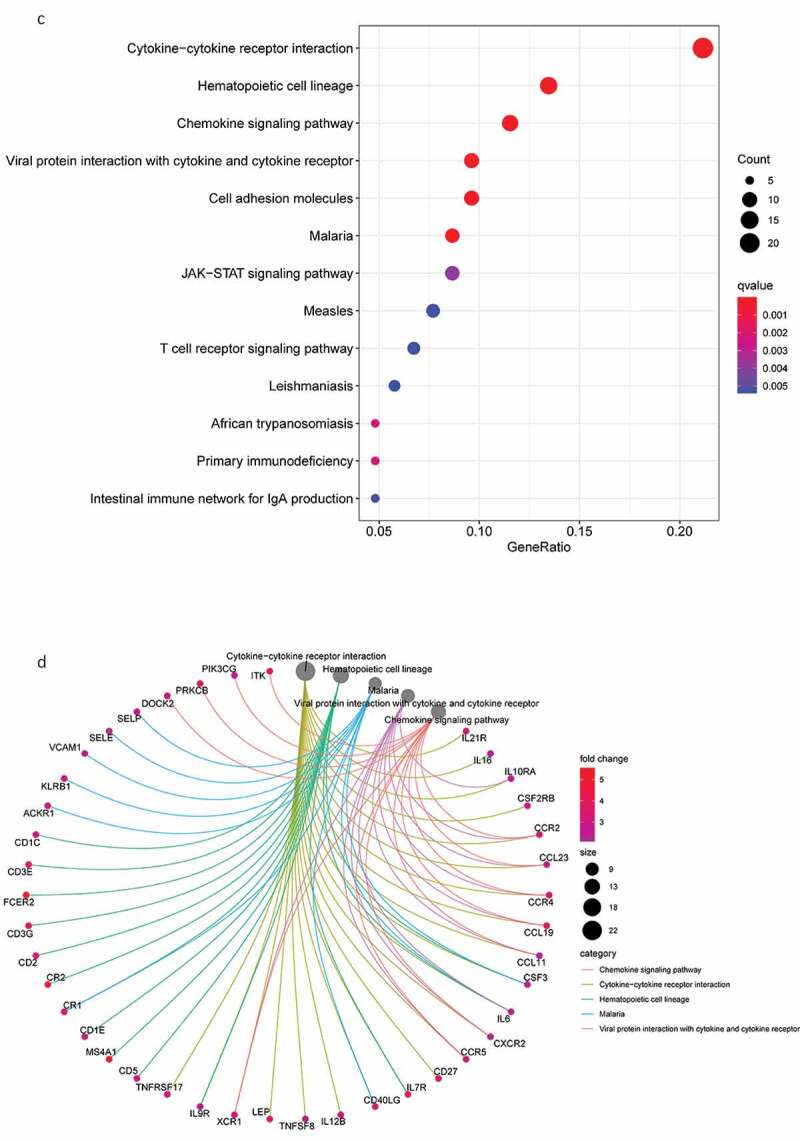
(Continued)

KEGG pathway analysis (Figure 3c) revealed 13 major signaling pathways, including viral protein interaction with cytokines and cytokine receptors, cytokine −cytokine receptor interactions, viral protein interaction with cytokines and cytokine receptors, hematopoietic cell lineage, malaria, and chemokine signaling pathways. Circos plots (Figure 3d) indicated that the KEGG pathways were mainly related to viral protein interaction with cytokines and cytokine receptors, malaria, cytokine−cytokine receptor interactions, hematopoietic cell lineage, and chemokine signaling pathways. In addition, these DEGs were significantly associated with immune regulation and thus require in-depth analysis.

### 3.4. Survival curves

To explore the prognostic association between the DEGs and the OS of patients with BC, we conducted survival analysis on the 226 overlapping DEGs, 66 of which were associated with OS (*P* < 0.05). Representative K-M curves for several genes are shown in Figure 4 and further details are provided in Supplementary Table 1A.

**Figure 4.**
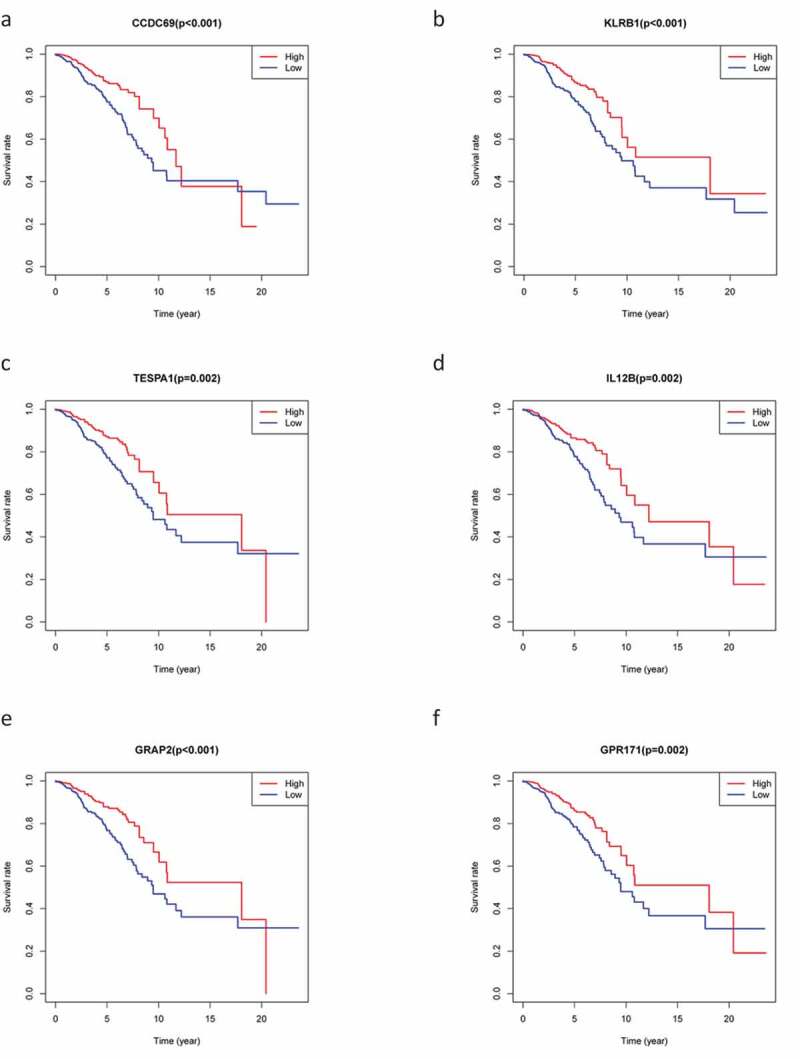
The survival curve shows the effect of the expression levels of 9 DEGs. In the figure, the overall survival time of patients with high gene expression (red line) was compared with the overall survival time of patients with low gene expression (blue line). P < 0.05 means the difference is significant. DEGs: differentially expressed genes

**Figure 4.**
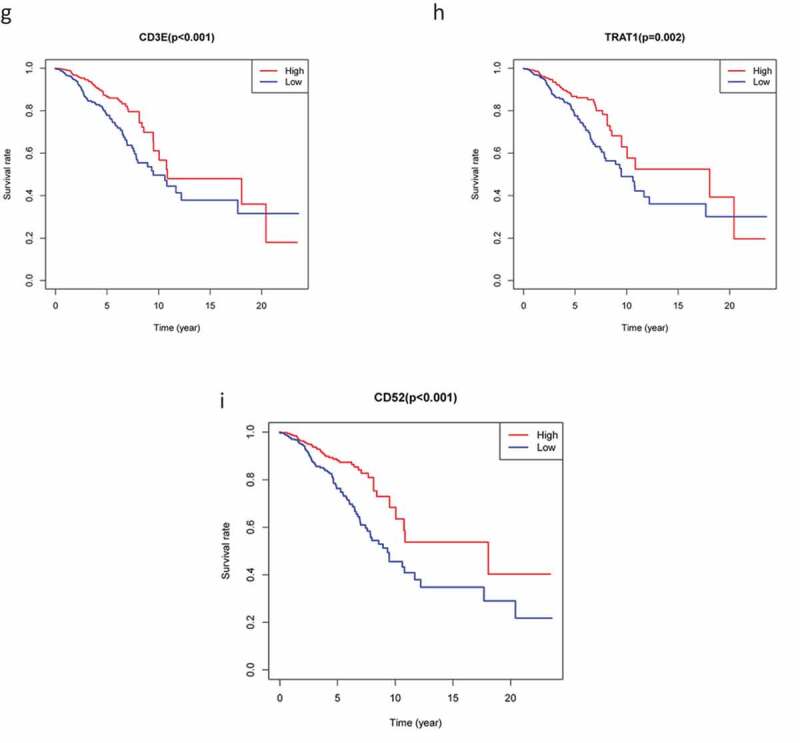
(Continued)

### 3.5. PPI network analysis

**Figure 5.**
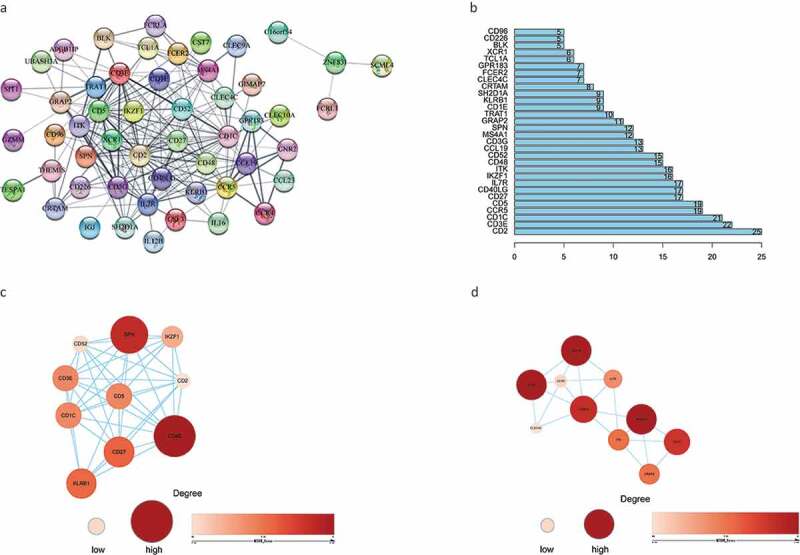
The PPI network was constructed, and two important modules were obtained by using Cytoscape. (a) PPI network of differentially expressed genes with integrated scores larger than 0.40. (b) The top 30 central genes identified in the PPI network. (c) CD48 module. (d) CD1E module. The darker the color of the node is, the greater the log2FC value of the gene expression and the larger the size of the node, the greater the number of edges between the gene and other genes. PPI: protein-protein interaction

A PPI network was constructed from the 66 genes that may have prognostic value using the STRING network analysis tool and core genes were analyzed using the CytoHubba plugin in Cytoscape software. The PPI network consisted of 66 nodes and 214 edges. The subnetwork with the most nodes and edges is shown in Figure 5a. We identified the top 30 central genes in the PPI network (Figure 5b), of which the top 10 nodes ranked by degree were *CD2, CD3E, CD1C, CCR5, CD5, CD27, CD40LG, IL7R, IKZF1*, and *ITK*. After the entire PPI network had been loaded into the cell landscape, two important hub genes, *CD48* and *CD1E*, were subjected to further analysis (Figure 5c,d). Notably, the majority of the key nodes in the PPI network consisted of proteins/genes involved in immune regulation.

### 3.6. Construction of a three-gene prognostic model for BC

To analyze the effect of various factors on OS, we constructed a Cox risk analysis model and simultaneously obtained a genetic signature related to the TME. Univariate Cox regression analysis of the 66 consensus genes identified 20 significant DEGs (*P* < 0.01; Table 1), while multivariate Cox regression analysis narrowed this down to just three key genes (Table 2).

**Table 1.** 

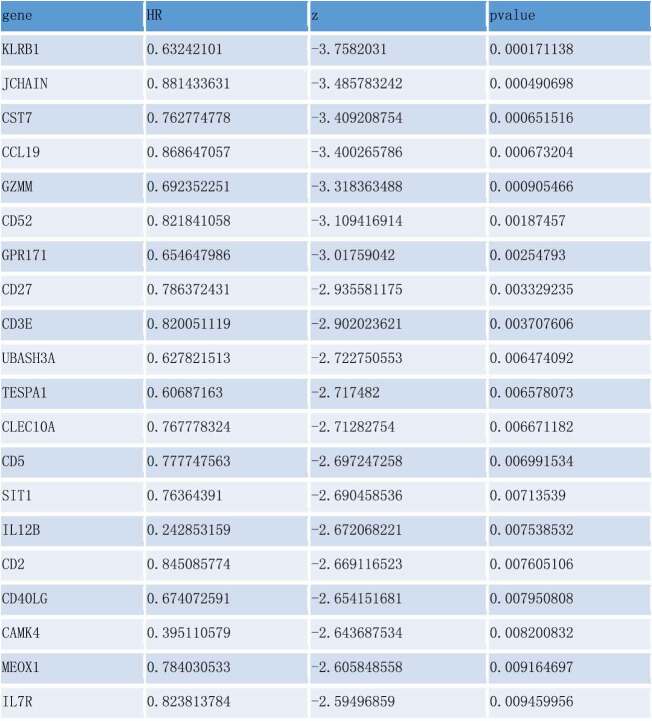

**Table 2.** 

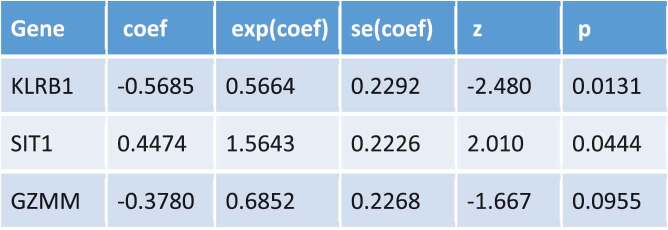

Risk score = (*KLRB1* × −0.5685) + (*SIT1 *× 0.4474) + (*GZMM* × −0.3780)

**Figure 6.**
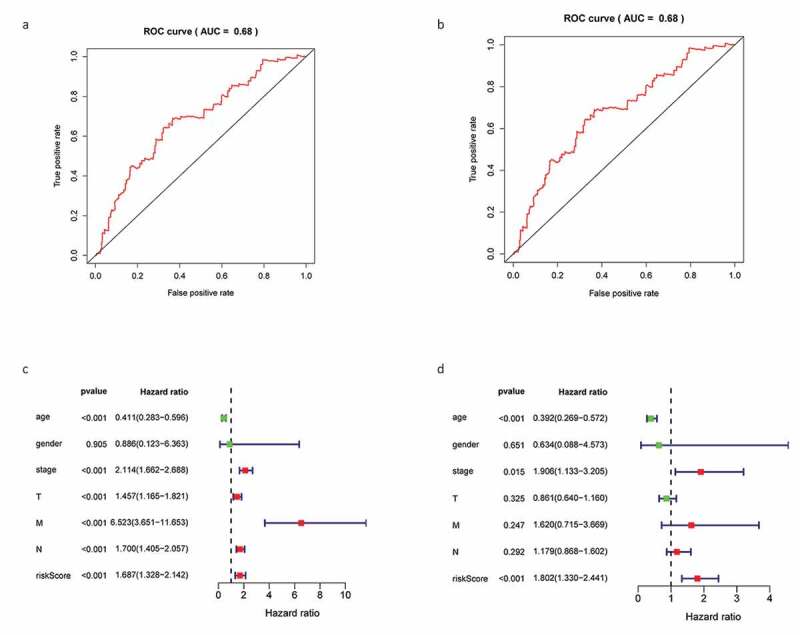
Construction gene signatures related to the TME through COX analysis. (a) K-M survival analysis showed differential expression between low- and high-risk groups. (b) Analysis of ROC curves. (c) Univariate Cox regression analyses of the common prognostic factors of BC. (d) The Multivariatemultiple Cox regression model analyses of the common prognostic factors of breast cancer. TME: the tumor tumor microenvironment. K-M: Kaplan–Meier analysis. ROC: receiver operating characteristics

K-M survival analysis and receiver operating characteristic (ROC) curves were used to describe the correlation between DEGs and the OS of patients with BC. K-M survival analysis revealed that high-risk patients displayed a significantly shorter survival time than low-risk patients (*P* = 0.008; Figure 6a). The area under curve (AUC) of the 3-year ROC curve was 0.68 (Figure 6b). Cox regression analysis revealed that risk score, age, and clinical stage could be used as prognostic factors for BC (Figure 6c,d) and that *KLRB1* and *SIT1* could be used as independent prognostic factors (*P* < 0.05).

### 3.7. GSEA

**Figure 7.**
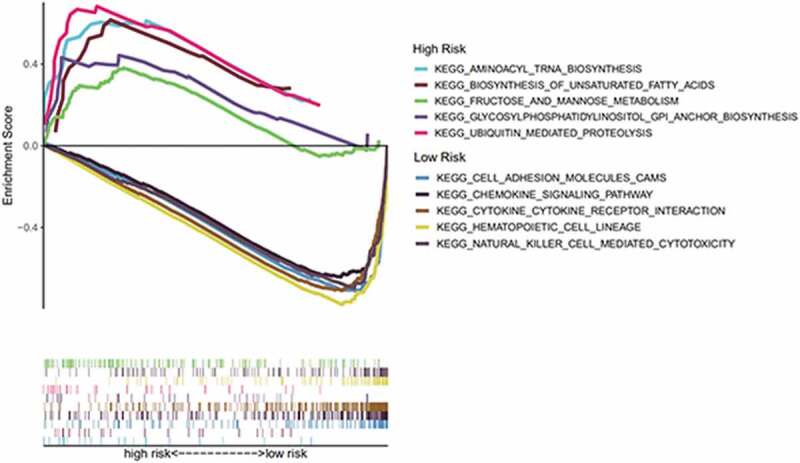
Through GSEA, we explored the difference in enrichment pathways between high- and low-risk groups. GSEA: gene set enrichment analysis

To further explore the difference in enrichment pathways between the high- and low-risk groups, we conducted GSEA. We found that pathways in the high-risk group were mainly related to metabolisms, such as aminoacyl tRNA biosynthesis, unsaturated fatty acid biosynthesis, fructose and mannose metabolism, glycosylphosphatidylinositol anchor biosynthesis, and ubiquitin-mediated proteolysis. Conversely, the low-risk group was associated with immune-related pathways, such as cell adhesion molecules, chemokine signaling pathways, hematopoietic cell line, natural killer cell-mediated cytotoxin, and cytokine–cytokine receptor interactions (Figure 7).

### 3.8. Immune cell infiltration analysis

Next, we determined the relationship between the screened genes and immune cell infiltration using the TIMER database. We found that *KLRB1, SIT1*, and *GZMM* (Figure 8a-c) were negatively correlated with tumor purity and positively correlated with the infiltration of B cells, CD4 T cells, CD8 T cells, neutrophils, macrophages, and dendritic cells.

**Figure 8.**
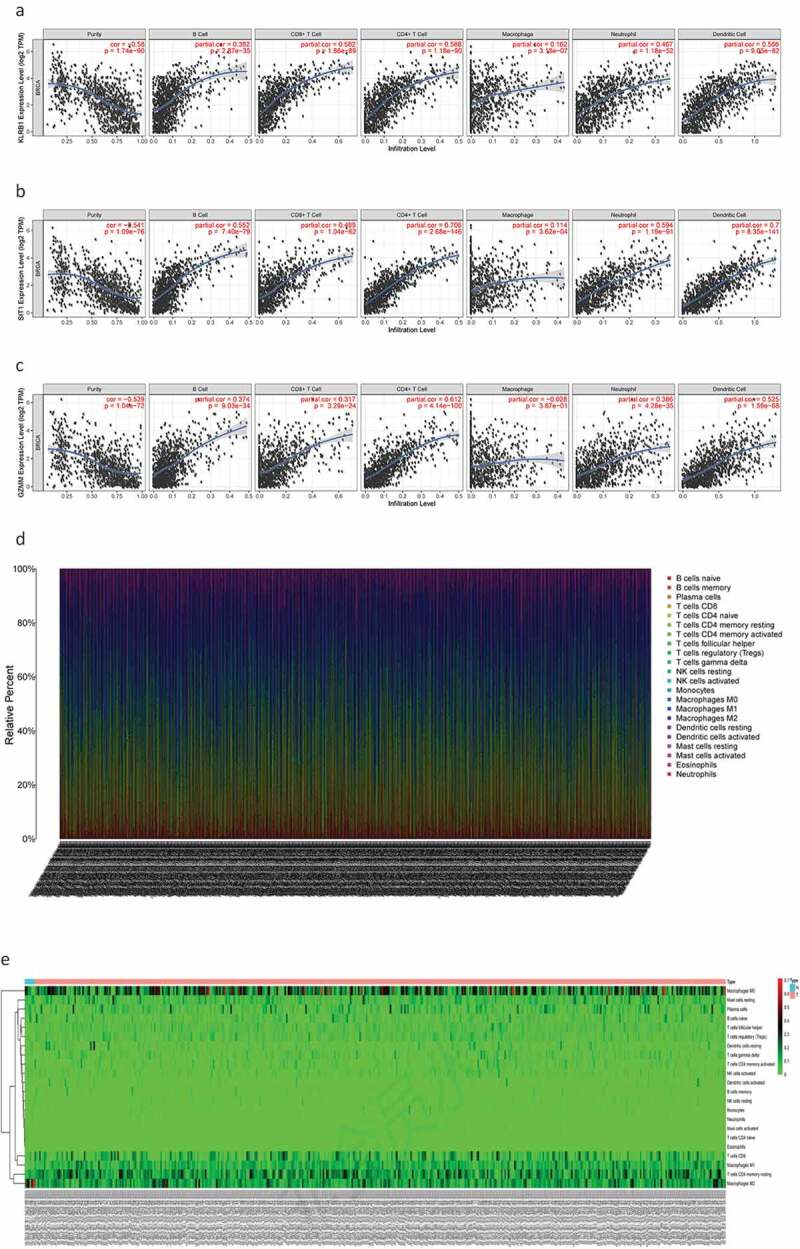
The relationship between the genes and immune cell infiltration. (a) *KLRB1*. (b) *SIT1*. (c) *GZMM*. (d) Bar plot showed the proportion of 22 immune cells with each sample. (e)The heat map showed the level of immune cell infiltration of each sample between normal tissues and BC tissues. (f) Correlation matrix of all 22 immune cells proportions. high is red, low is blue, and the same association levels is white. (g) Violin plot showed the proportions of 22 immune cells between normal tissues with BC tissues

**Figure 8.**
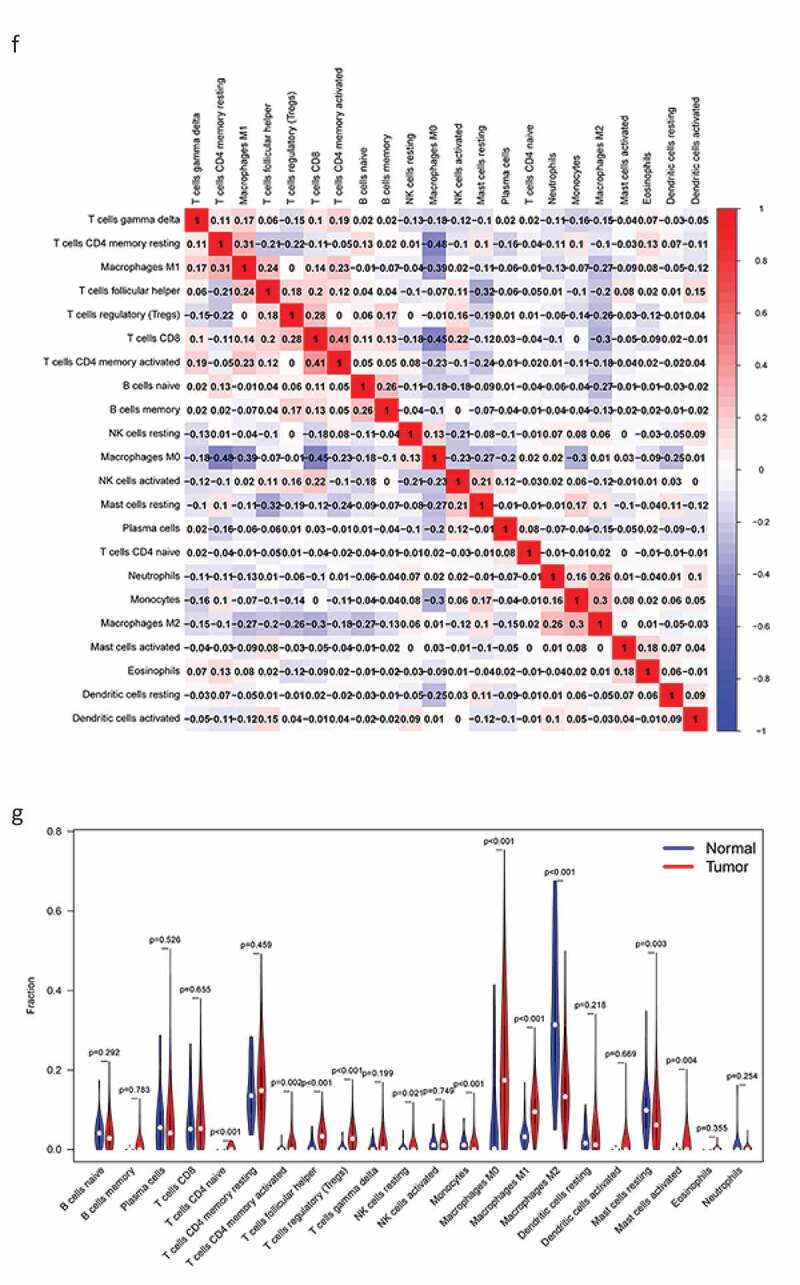
(Continued)

Therefore, we analyzed differences in immune infiltration between the low- and high-risk groups for 22 immune cells using Cibersort. First, we presented the proportion of each immune cell in all samples using a bar plot (Figure 8d) and then used a heat map to compare the levels of immune cell infiltration between normal and BC tissues (Figure 8e). Low to moderate correlation was observed in various immunocyte subpopulations (figure 8f) and the violin plot (Figure 8g) revealed that BC tissues displayed a higher proportion of activated CD4 memory T cells, follicular helper T cells, Tregs, resting natural killer (NK) cells, monocytes, M0, M1, and M2 macrophages, resting mast cells, and mast cells than normal tissues. Conversely, BC tissues displayed lower proportions of naïve and memory B cells, plasma cells, CD8T cells, resting CD4 memory T cells, gamma delta T cells, activated NK cells, resting and activated dendritic cells, eosinophils, and neutrophils. In addition, we compared immune cell infiltration in the high- and low-risk groups (Supplementary Figure 2).

### 3.9. HPA

**Figure 9.**
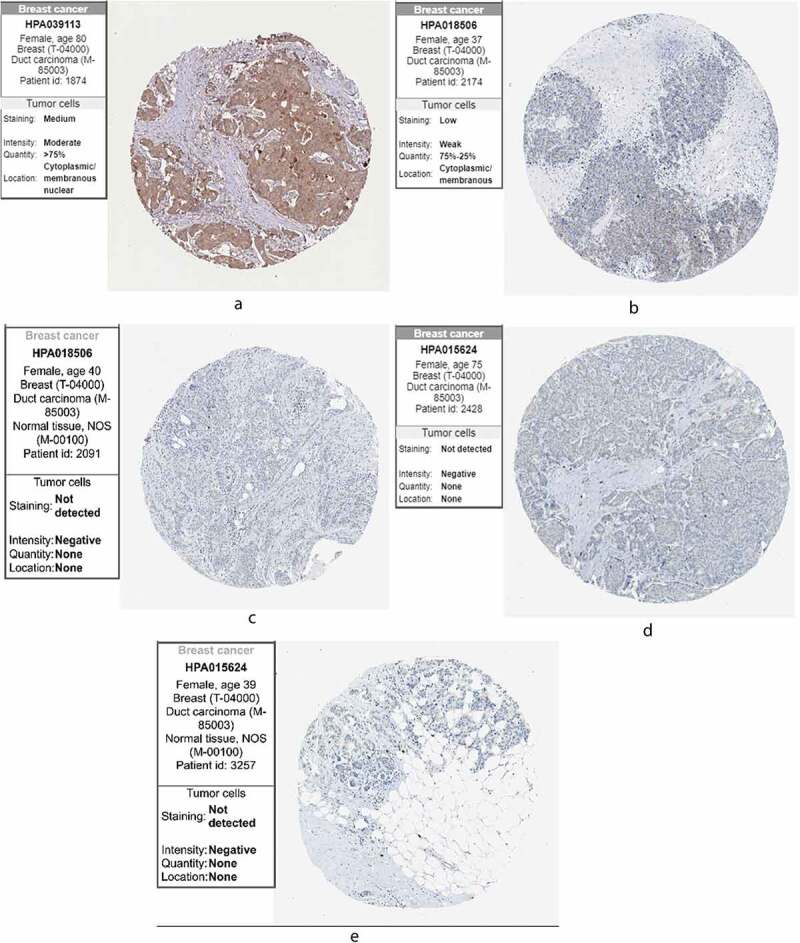
The expression of the genes in breast cancer and normal tissues in HPA. (a) *KLRB1*. (b) *SIT1*. (c) *GZMM*. HPA: the Human Protein Atlas

Finally, we compared the protein expression of the three core genes in control and BC tissues from the HPA, finding that KLRB1 (Figure 9a) was moderately highly expressed in BC tissues. SIT1 (Figure 9b) displayed low-medium expression, while it was negative in the adjacent tissues. GZMM (Figure 9c) showed negative expression and was also negative in the adjacent tissues. This may be due to the small sample size of the HPA database.

## 4. Discussion

BC is the most common cancer among women and is the second most commonly diagnosed cancer worldwide [17]. Increasing evidence has shown that the TME (including tumor-infiltrating immune cells) supports the growth and development of BC and affects tumor invasion, metastasis, and drug sensitivity [18]. Although various components of the TME have been shown to promote cancer progression, such as immune cells, soluble factors, and extracellular matrix alterations, the relationship between TME-related genes and cancer prognosis remains unclear [19]. TCGA database mining and analysis are commonly used to predict the prognosis of patients with cancer [20]; therefore, we used the TCGA database to identify TME-related genes with a significant effect on BC prognosis, analyze the biological processes and signal transduction pathways of related DEGs, and evaluate the predictive ability of gene signatures.

First, we obtained stromal, immune, and ESTIMATE scores using the ESTIMATE algorithm and then investigated the relationship between these scores and the clinical information for 1,049 samples. A significant negative correlation was observed between clinical stage and immune score, while patients with BC that had higher immune scores had a longer OS than those with low scores and the stromal score did not significantly differ. These results indicate that immune cells may play a key role in the TME of BC. Consistently, previous studies have shown that infiltrating immune cells affect the biological and clinical processes underlying BC [21]and that recurrence and disease-specific mortality rates increase as patient age increases [22].

Next, we screened 226 DEGs by comparing the expression levels in the high- and low-scoring groups, with GO and KEGG pathway analyses revealing that these DEGs are related to the activation and differentiation of immune cells. In addition, KEGG pathway analysis indicated that these DEGs are mainly involved in immune regulatory pathways. These findings are consistent with previous studies which showed that activated T cells in the TME can promote tumor cell death [23] and that chemokines and their receptors play a key role in determining the metastatic destination of tumor cells [24].

We then constructed a PPI network from 66 DEGs that were closely related to prognosis and screened genes with the highest connectivity, identifying *CD2, CD3E, CD1C, CCR5, CD5, CD27, CD40LG, IL7R, IKZF1*, and *ITK* as the top 10 nodes ranked by degree. According to previous reports, *CCR5* expression in human BC is associated with a poor prognosis [25], while some of the other genes identified are involved in immune regulation. For instance, *CD2* is known to mediate T and NK cell activation by interacting with *CD58* [26], while the CD1c+ DC subset is a major inducer of the CD4 T cell response [27]. Moreover, CD5 is recognized as an important marker of malignant T cells that is expressed in almost all normal T cells [28]. CD27 and its ligand CD70 are involved in the regulation of cellular immune responses to cancer and also enhance T cell proliferation and memory-cell formation [29]. Furthermore, T lymphocytes have been found to lack *CD40* ligand expression due to *CD40LG* gene inactivation [30]. In BC, *IL-7* promotes tumor growth by activating the *JAK1/3-STAT5* and *PI3K/AKT* pathways [29], while the *IKZF1* gene plays important regulatory roles in lymphogenesis [31]. Finally, *ITK* is a member of the TEC kinase family that is involved in regulating T cell receptor signaling and T cell differentiation [32].

Consequently, we constructed a prognostic model and analyzed these 66 DEGs using Cox proportional hazard regression analysis to obtain three key genes. We found that risk score, age, and clinical stage can be used as prognostic factors for BC, while *KLRB1* and *SIT1* can be used as independent prognostic factors. Consistently, the expression of *KLRB1* (encoding *CD161*) has been shown to reflect tumor-associated leukocytes [33], while *CD161* is generally regarded as a marker of NKT cells [34]. In addition, previous studies have reported that the percentage of *CD161*(+) NKT cells in tumor and breast lymph nodes is significantly higher than in normal tissues [35]. *SIT* is a transmembrane adapter protein that participates in receptor signal transduction in immune cells, exists in both T and B cell subpopulations [36], and is an important regulator of TCR-mediated signaling, including T-cell homeostasis and tolerance [37]. Therefore, these two key prognostic genes are closely related to immunity.

GSEA revealed key differences between the high- and low-risk groups; in particular, the low-risk group was mainly associated with immune-related pathways, while the high-risk group was mainly related to metabolism. Therefore, we investigated the potential molecular mechanisms related to immune and metabolic pathways in the BC microenvironment by studying the types and proportions of infiltrating immune cells in the BC microenvironment. TIMER analysis revealed that *KLRB1, SIT1*, and *GZMM* were negatively correlated with tumor purity and positively correlated with the infiltration of B cells, neutrophils, macrophages, CD4 T cells, CD8 T cells, and dendritic cells. Therefore, we compared the infiltration of 22 immune cell types in the high- and low-risk groups. CIBERSORT revealed that the immune cell subpopulations were low to moderate and that BC tissues contained higher proportions of activated CD4 memory T cells, follicular helper T cell, Tregs, resting NK cells, monocytes, M0, M1, and M2 macrophages, as well as resting and activated mast cells. Conversely, BC tissues displayed lower proportions of naïve and memory B cells, plasma cells, CD8T cells, resting memory CD4 T cells, gamma delta T cells, activated NK cells, resting and activated dendritic cells, eosinophils, and neutrophils. Tregs have been shown to support a hostile TME to promote BC progression [38] and play an important role in inhibiting cancer progression to suppress cancer-promoting inflammatory processes [39]. Tumor-associated macrophages (TAMs) can be divided into M1 inflammatory and M2 anti-inflammatory subgroups that express inflammatory or non-inflammatory chemokines, respectively. TAM infiltration in the TME contributes toward cancer progression and metastasis via several pathways, including stimulating angiogenesis, tumor growth, and cellular migration and invasion [40]. Indeed, M2 macrophages (*CD163*-positive) are associated with progression to invasive BC [41]. Finally, we verified the protein expression of the three core genes using the HPA database, finding that *KLRB1* was moderately highly expressed in BC tissues, *SIT1* was expressed at low-medium levels, and *GZMM* was negatively expressed. Together, these results further verify the findings of our analyses.

Unfortunately, this study also has several shortcomings. Firstly, we only selected certain types of BC (lobular and ductal tumors); therefore, our survival analysis was not comprehensive and complete. Secondly, this study lacked the necessary experiments to verify the identified genes. In conclusion, this study based on the big data analysis of TCGA database adds to the description of the gene characteristics of the TME in BC and identifies relevant influential pathways.

## Supplementary Material

Supplemental Material

Supplemental Material

## Highlights

- KLRB1 and SIT1 genes can be used as independent prognostic factors of BC microenvironment.
- The prognosis of BC microenvironment is related to the immune pathway.
