# Supplementary figures and images for: Bioinformatics analysis to screen key prognostic genes in the breast cancer tumor microenvironment
Source: Bioengineered. 2020 Nov 8;11(1):1280–300. doi: 10.1080/21655979.2020.1840731 (PMC8291857; doi:10.1080/21655979.2020.1840731)

Supplymentaty Figure 1

A B


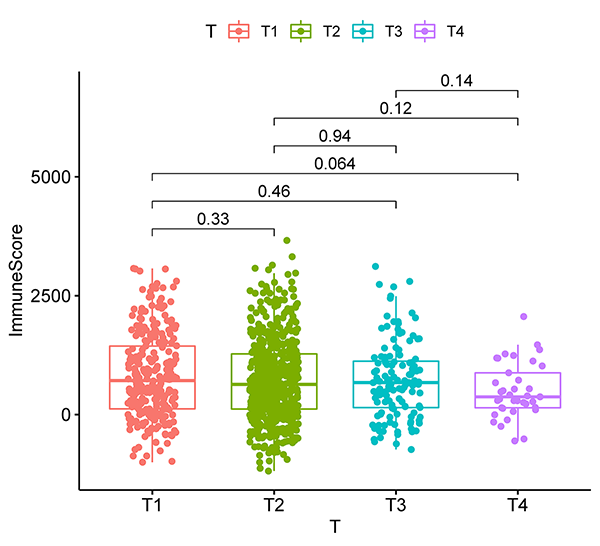

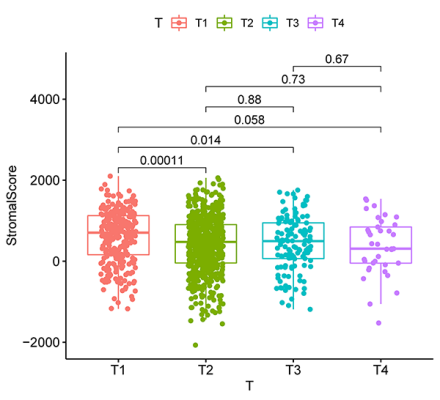


C D


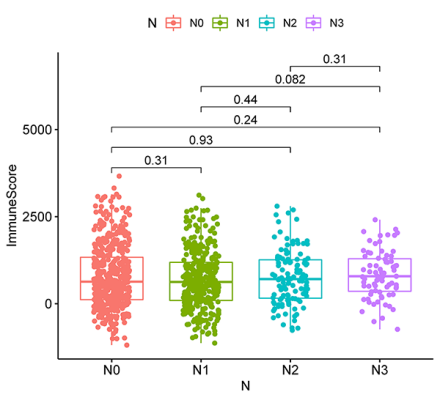

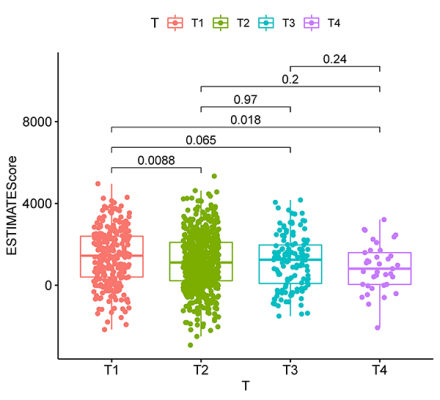


E F


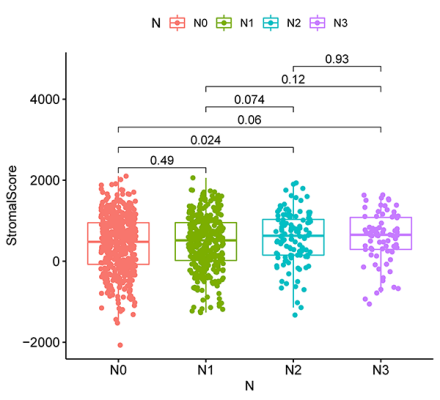

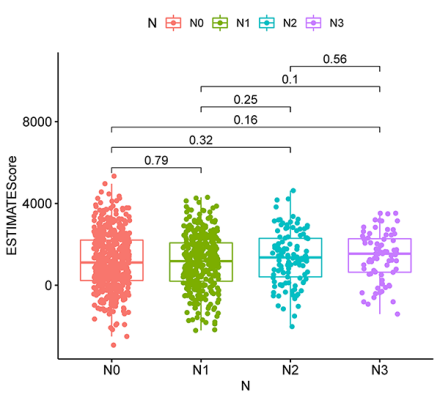


G H


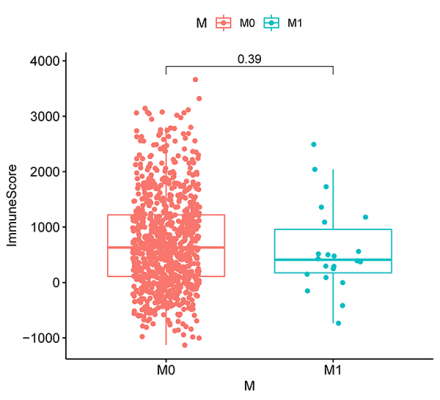

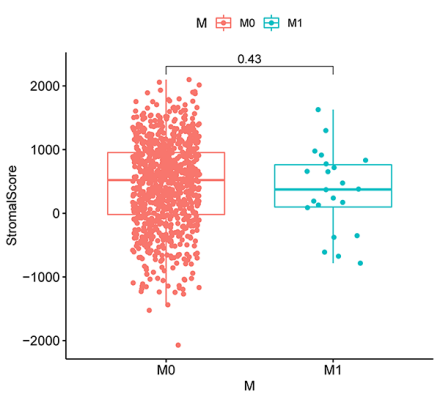


I J


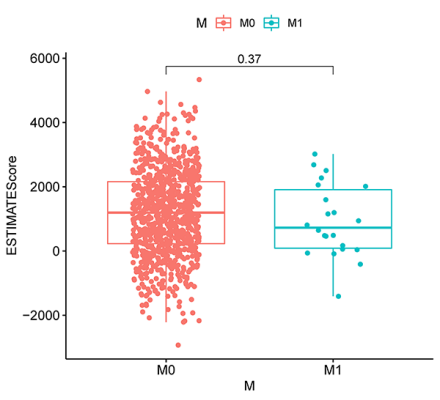

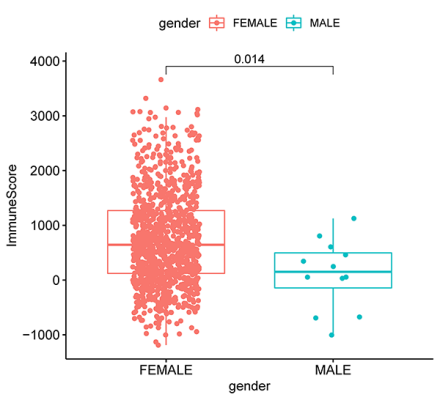


K L


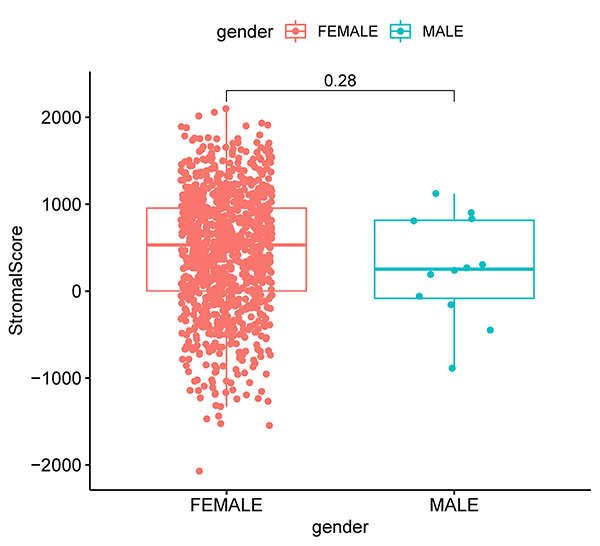

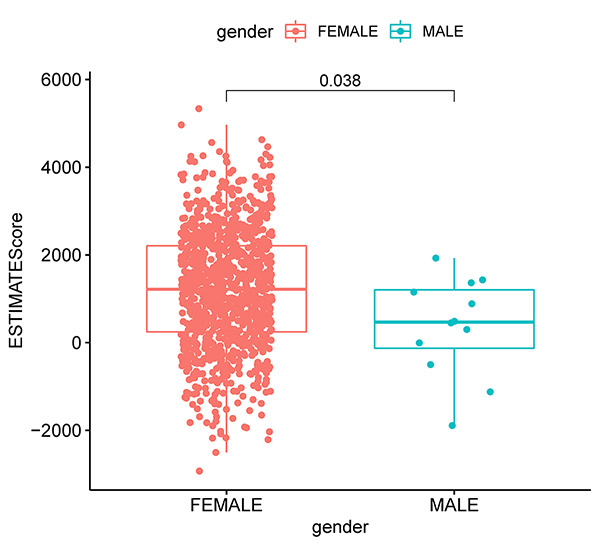


Supplymentaty Figure 2

A B


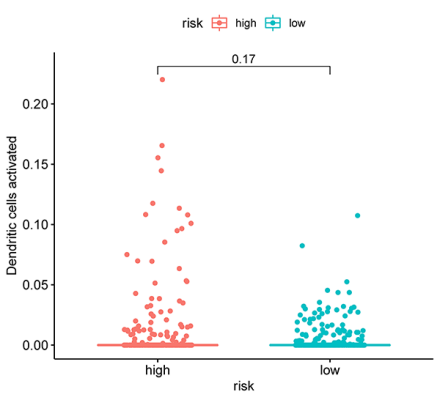

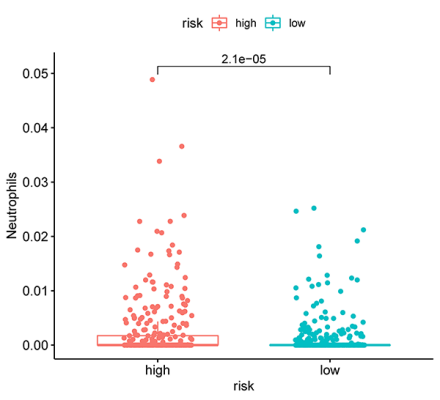


C D


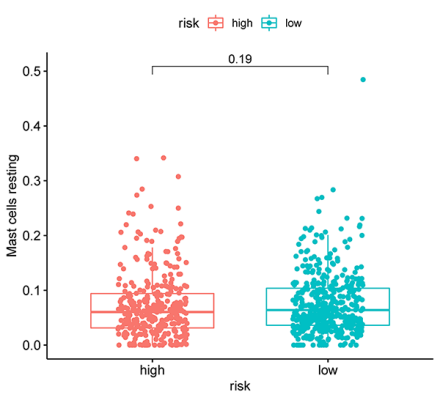

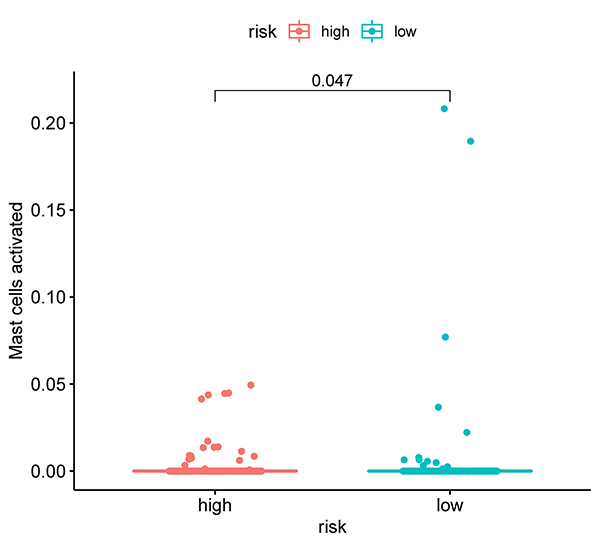


E F


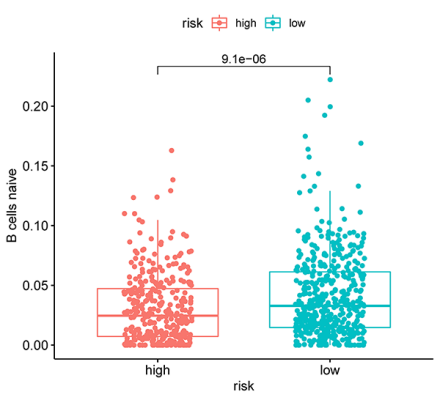

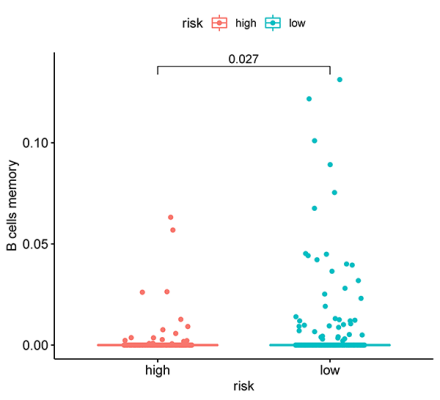


G H


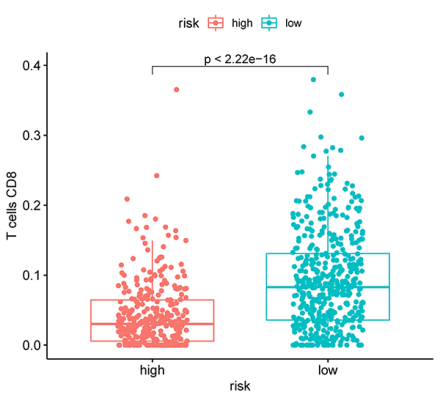

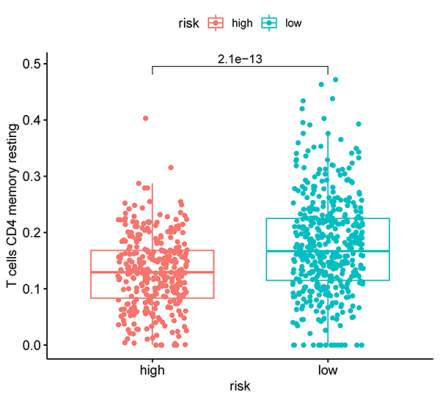


I J


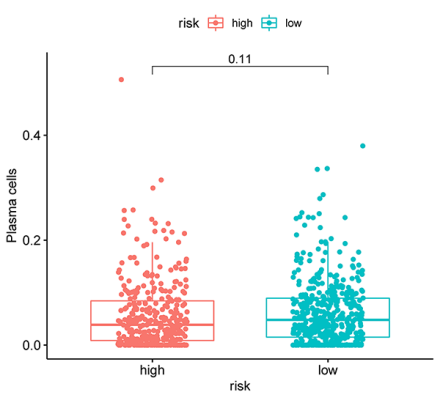

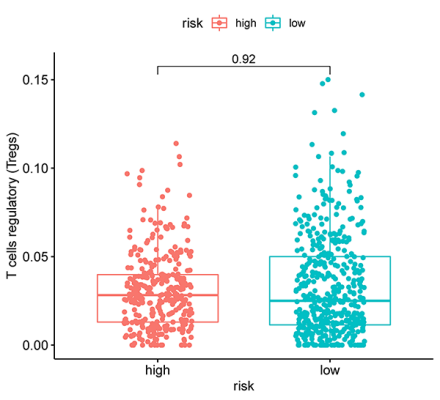


K L


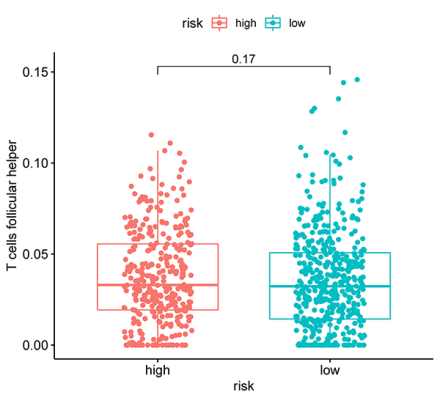

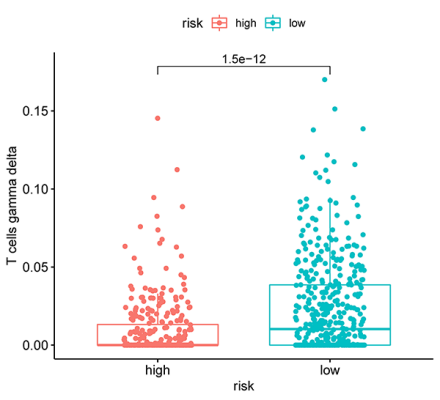


M N


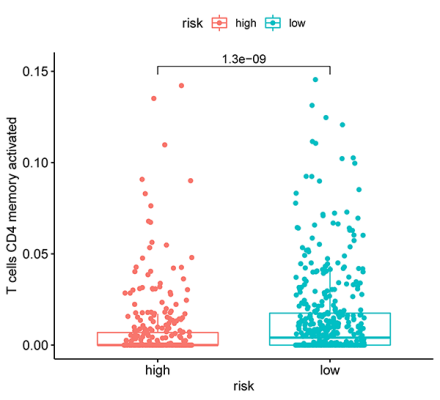

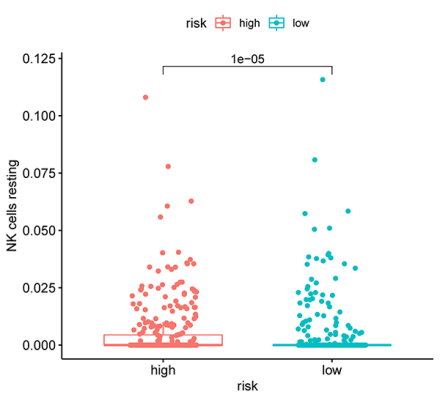


O P


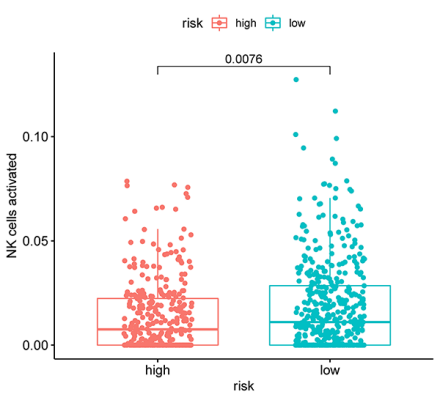

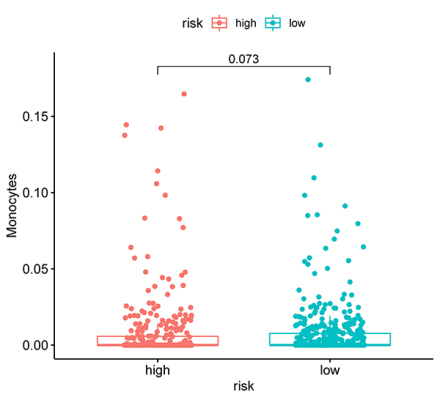


Q R


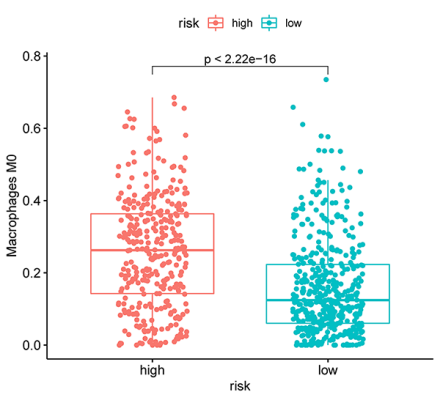

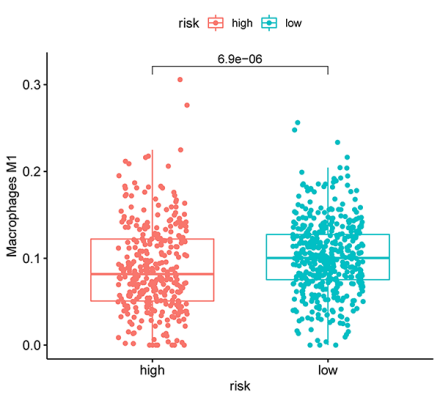


S U


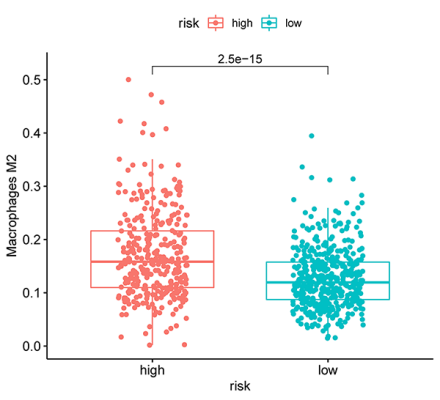

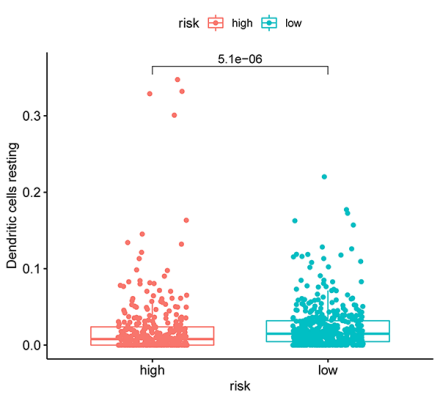

Supplement: Supplemental Material [file KBIE_A_1840731_SM8721.docx]
