# Supplementary material for: Bioinformatics analysis to screen key prognostic genes in the breast cancer tumor microenvironment
Source: Bioengineered. 2020 Nov 8;11(1):1280–300. doi: 10.1080/21655979.2020.1840731 (PMC8291857; doi:10.1080/21655979.2020.1840731)
Supplement: Supplemental Material [file KBIE_A_1840731_SM8706.docx]

| gene | pvalue |
| --- | --- |
| CD52 | 6.57E-05 |
| KLRB1 | 0.000349287 |
| GRAP2 | 0.000472364 |
| CCDC69 | 0.000498689 |
| CD3E | 0.000844392 |
| TESPA1 | 0.001548929 |
| IL12B | 0.00172226 |
| TRAT1 | 0.00179476 |
| GPR171 | 0.001902962 |
| CD5 | 0.002317373 |
| CNR2 | 0.002350352 |
| CD48 | 0.002759456 |
| SLFN12L | 0.003465792 |
| DTHD1 | 0.003578742 |
| CD40LG | 0.004171585 |
| FCRLA | 0.004251493 |
| SCML4 | 0.005313749 |
| CD1C | 0.0057198 |
| CAMK4 | 0.005966017 |
| CD96 | 0.006429202 |
| MS4A1 | 0.00759858 |
| BLK | 0.007629851 |
| TCL1A | 0.007652366 |
| SH2D1A | 0.008345327 |
| JAML | 0.00881449 |
| CD2 | 0.009077027 |
| PYHIN1 | 0.010692002 |
| CCL19 | 0.011630249 |
| CST7 | 0.011644393 |
| CLEC10A | 0.01200865 |
| JCHAIN | 0.012634767 |
| ITK | 0.012664012 |
| C1S | 0.013243615 |
| UBASH3A | 0.015442859 |
| CD226 | 0.015480391 |
| IL16 | 0.015605738 |
| CCR5 | 0.015680593 |
| FCRL5 | 0.017083464 |
| IKZF1 | 0.01712278 |
| CLEC4C | 0.018085608 |
| GZMM | 0.018785903 |
| APBB1IP | 0.018897892 |
| IL7R | 0.020490214 |
| SIT1 | 0.022574789 |
| FCRL1 | 0.02414719 |
| CCL23 | 0.024906853 |
| GIMAP7 | 0.025013358 |
| CD3G | 0.027505535 |
| CRTAM | 0.028744547 |
| SAMD3 | 0.028981133 |
| CD27 | 0.030768587 |
| CLEC9A | 0.03226866 |
| XCR1 | 0.035037081 |
| CCR4 | 0.035397353 |
| TEX11 | 0.035980246 |
| FCRL3 | 0.036257386 |
| SPN | 0.037996734 |
| TREML2 | 0.041919401 |
| FCER2 | 0.042680477 |
| GPR183 | 0.044002231 |
| C16orf54 | 0.045301486 |
| THEMIS | 0.045659997 |
| ZNF831 | 0.04656194 |
| CD1E | 0.046840766 |
| CSF3 | 0.048223728 |
